# Urgent response is needed to address the stimulant-fentanyl related overdose crisis

**DOI:** 10.3389/fpsyt.2026.1769408

**Published:** 2026-03-17

**Authors:** Rosalina Mills, Oluwole Jegede, Srinivas B. Muvvala

**Affiliations:** 1 Department of Psychiatry, Yale School of Medicine, New Haven, CT, United States; 2 Connecticut Mental Health Center, New Haven, CT, United States

**Keywords:** contingency management (CM), drug contamination/prevention and control, harm reduction (HR), opioid use disorder, overdose prevention, safe supply, stimulant use disorder, structural competency

## Introduction

The fourth wave of the opioid epidemic is characterized by the contamination and/or intentional use of stimulants (cocaine, methamphetamine), synthetic opioids (fentanyl and its analogs), and other synthetic drugs. This emerging pattern of use has resulted in a marked increase in overdose deaths across the United States (US) (1, 2). Due to unregulated drug supplies, the risk of encountering synthetic opioid adulterants and unintentional overdose is growing for people who use unprescribed stimulants. US estimates reflect a 5.9%-15% prevalence of unregulated stimulant samples containing fentanyl (3–5). This is especially evident in the northeast, where cocaine has been the most commonly co-involved substance with fentanyl since in 2019 (6). Connecticut, for example, saw a 9.3% increase in cocaine-related deaths between 2021-2022 (7). Stimulant use overall is also increasing among Americans. Estimates show a 43% increase in methamphetamine use over four years, between 2015-2019 (8). Between 2021-2022, the prevalence of stimulant use increased 8.63%; from 9.4 million to 10.2 million Americans (9, 10). Methamphetamine use disorder among Black individuals in particular increased 10-fold over a four-year period from 2015-2019 (8).

This epidemic has also shown a relatively recent racial shift, namely, a widening disparity in overdose mortality rates among Black and American Indian/Alaskan Native (AI/AN) populations (1, 11). Starting around 2020, the rise in overdose mortality has disproportionately impacted Black and AI/AN populations (1). This trend has been shown to be due to many documented causes at the individual, interpersonal, organizational, and policy levels (12). White Americans are more likely to be legally prescribed medications for pain and opioid use disorder, while minoritized people must more often resort to illicit or unregulated sources, thus increasing the risk of variability in their drug supply (13). These treatment disparities often arise from stigma and implicit bias, manifested as inaccurate beliefs that minoritized people have higher pain tolerance, further driving inequities in care (1). Convergent data shows that stimulant-opioid co-use is higher among minoritized populations in the US (13), thus increasing the risk of accidental overdose, as community supplies are often inconsistent in their ingredients, thus exposing people who use drugs to harm. Consistent epidemiologic data reflects these worsening disparities and highlights the urgency for robust and targeted population-level and structural intervention.

## Intervention and prevention strategies

Multilevel approaches are needed to address the escalating overdose epidemic among people who use stimulants, especially considering the disproportionate effect of the overdose crisis on historically minoritized communities. If we are to effectively tackle the ongoing stimulant-opioid co-use crisis and move toward more equitable outcomes, we must prioritize multisectoral and interdisciplinary collaboration, community awareness, policy, and institutional reforms and integrate the following approaches into structural, policy, and clinical care: 1. Incorporating structural competency into addiction provider education; 2. Scaling evidence-based interventions such as contingency management (CM); 3. Including peer, community, and family engagement; 4. Expanding harm reduction services; 5. Optimizing diagnosis and access to pharmacologic interventions; and 6. Appropriately implementing cultural adaptations of evidence-based interventions and contexts.

### Structural competency in provider education

Structural competency (SC) education advances traditional approaches to healthcare professional education by emphasizing a cultural understanding of the experiences of people with substance use disorders (SUDs), and increasing awareness of systemic upstream forces that influence health outcomes (14). SC provides a culturally appropriate formulation for individuals with substance use disorders, thereby helping providers reduce implicit bias and work from an equity-based perspective. Addiction clinicians and practitioners could effectively incorporate community family, and other stakeholders (e.g., religious institutions) in treatment (15, 16), as incorporating cultural values into SUD (including stimulant use disorder (StUD)) treatment improves outcomes, especially when providers exhibit cultural humility.

### Implementing, scaling, and evaluating contingency management

From an intervention perspective, implementing, scaling, and evaluating evidence-based approaches such as Contingency Management (CM) for StUD is needed to help address the stimulant-opioid toxicity crisis (17–20). Increasing the availability, accessibility, and affordability of CM could be a vital strategy in stemming this epidemic.

CM has demonstrated promising evidence for StUD treatment and thereby carries the potential to mitigate overdose risk in the fourth wave of the opioid epidemic (21–23); but its implementation has been severely limited. CM involves the delivery of tangible rewards (e.g., gift cards, vouchers, etc.) in exchange for participants providing proof of abstinence from stimulants (most commonly, a negative urine test) (19). Decades of evidence from randomized controlled trials have presented definitive evidence of CM as the top choice for the management of StUD, either alone or in conjunction with pharmacotherapies (19, 24–30).

Despite the documented efficacy of CM in clinical trials, very few US-based institutions have adopted this strategy outside the VA system. Healthcare professionals are largely unaware and/or unfamiliar with CM as a viable approach to StUD (22, 31, 32). Barriers to CM implementation include limitations of insurance coverage, regulatory obstacles, and lack of funding that have made it difficult for StUD patients to access CM (22). For example, California is currently the only state to include CM in their Medicaid program; they have renamed their CM programs as Recovery Incentives Programs (33). All other states are limited to a maximum of $75 per participant for the implementation of CM treatment, which is far below the evidence base to sustain effectiveness of a CM program (22). Organizations participating in the delivery of CM include SUD treatment centers, hospitals, community health clinics, corrections facilities, and occasionally, fire departments (22). Integrating CM into addiction treatment models, primary care and mental health can potentially revolutionize treatment outcomes for people who use stimulants.

Emerging literature suggests the importance of cultural adaptation of CM for underserved and minoritized populations (34). If not carefully designed through an equity lens, there remains a potential for inequitable CM implementation. CM programs for minoritized communities must be developed with input from people with lived experience and must focus on dignity, autonomy, and acknowledgment of real-world barriers to participation (35). Additional critical implementation outcomes must also be considered including uptake, adherence, retention, and acceptability when designing CM programs for minoritized patient populations (35). Consideration of health behaviors, cultural, and environmental factors are vital while adapting CM (28, 35). Cultural adaptation has been shown to be of critical importance for implementing CM; for example, in AI/AN populations with alcohol use disorder, appropriate cultural adaptations of CM resulted in increased treatment participation and sustainability (36, 37). There remains however a dearth of literature on cultural adaptations of CM for StUDs.

### Community, peer, and family engagement

Community, peer, and family engagement are vital in supporting prevention and intervention efforts for the recovery journeys of individuals with disordered stimulant/opioid co-use. Recovery models involving the collaboration of healthcare professionals, local communities, and church leaders (38, 39) have been particularly effective at improving recovery outcomes, particularly for minoritized populations. Family-based programs such as Community Reinforcement and Family Training (CRAFT) (40) have been developed for the engagement of families of individuals with substance use disorders. Including family and community members in recovery journeys as part of a multipronged approach to prevention and recovery may help address overdose risk among people who co-use stimulants and opioids.

### Pharmacotherapy and harm reduction

Public health approaches such as harm reduction have been proven to reduce drug-related overdose mortality (41, 42). While there are currently no Food and Drug Administration (FDA)-approved pharmacotherapies for StUD, off-label medications such as bupropion, bupropion-naltrexone combination, topiramate, and mirtazapine have shown some benefit and can be utilized in StUD treatment (24). While evidence regarding the effectiveness of long-acting psychostimulants for StUD is mixed, this approach has also shown some positive evidence in StUD patients, particularly those with cocaine use disorder, and can be offered as part of wraparound harm reduction services (43–48). The use of prescribed psychostimulants for StUD may require close monitoring and ongoing evaluation to mitigate misuse risks or emergent psychotic decompensation in those with co-occurring psychotic disorder (24, 49).

Additionally, screening, diagnosis, and treatment for individuals with co-occurring attention deficit hyperactivity disorder (ADHD), given the prevalence of ADHD and StUD co-morbidity, is recommended to reduce the social and functional burden of disease, multimorbidity, and mortality among these patient populations. A multi-modal approach combining pharmacological (stimulant or non-stimulant) and non-pharmacological approaches such as integrated cognitive behavioral therapy is recommended for treatment of comorbid ADHD and StUD (50).

Similarly, medications for opioid use disorder (MOUD) such as buprenorphine, methadone, and naltrexone have been shown to reduce overdose risk in people with opioid use disorder (OUD) and could be prescribed to people with co-occurring StUD and OUD (51). Still, evidence on the effectiveness of this treatment on this patient population is sparse; additional research is needed to develop and implement sustainable treatment pathways for patients with stimulant and opioid co-use (52).

Other harm reduction approaches to prevent overdose in people who co-use stimulants and opioids involve the distribution and utilization of test strips for adulterants such as fentanyl and xylazine. Checking drugs for adulterants in the drug supply enables informed decision-making regarding continued use, safety protocols (carrying naloxone, using in the presence of others), and dosage (53, 54). Likewise, expanding access to drug checking services to detect adulterant presence and levels in drug samples helps individuals reduce overdose risk (55). Recent data has also demonstrated a distinct need for culturally adaptive harm reduction interventions for racialized populations, who often have limited access to harm reduction and preventive resources (56). Developing equitable implementation strategies to adapt and scale harm reduction measures will help increase access and utilization of these services.

Increasing access, availability, and distribution of naloxone, the opioid antagonist that reverses opioid overdose, is another vital step in combating overdose death. Policy experts, the Centers for Disease Control (CDC), and professional clinical guidelines have encouraged routine carrying of naloxone for people who use stimulants considering the contamination of the unregulated drug supply with high potency synthetic opioids (57, 58). Finally, amending targeted outcomes may also be a useful approach in the treatment of clients with StUD. Assessing abstinence as the sole indicator of treatment success may overlook improvement in other domains, such as increases in behaviors that reduce overdose risk (e.g., carrying naloxone); reductions in higher-risk behaviors (e.g., solitary use, intravenous use); and improved quality of life (41).

## Discussion

Healthcare delivery and public policy must focus on implementation and dissemination approaches to scale evidence-based interventions using equity-based frameworks that center social determinants of health, culturally appropriate, and multifocal approaches. Different approaches to reducing overdose deaths from unregulated stimulant use may work for different populations, suggesting that culturally tailored interventions may be vital for increasing access and utilization of resources aimed at reducing overdose deaths. Implementation frameworks must center social determinants of health, and policy approaches must work within equity frameworks to reach minoritized populations.

While preliminary evidence supports MOUD, CM, and community-led overdose prevention measures for co-occurring stimulant and opioid co-use, these strategies lack the longitudinal data and robust clinical trials necessary for systematic, large-scale implementation. To adequately address the current wave of the overdose epidemic, more research is needed on the most effective methods to reduce overdose deaths in people with stimulant and opioid co-use.

Adapting and scaling evidence-based approaches such as harm reduction and CM to underserved and minoritized populations must be a priority in organizational and individual psychiatric practice. A more global approach that includes a focus on polysubstance use, culture-based adaptations of available treatments, and multidisciplinary approaches are likely to reduce overdose mortality within communities using stimulants combined with fentanyl and other synthetic opioids.
